# Supplementary figures and images for: Induction of systemic immunity through nasal-associated lymphoid tissue (NALT) of mice intranasally immunized with Brucella abortus malate dehydrogenase-loaded chitosan nanoparticles
Source: PLoS One. 2020 Feb 6;15(2):e0228463. doi: 10.1371/journal.pone.0228463 (PMC7004331; doi:10.1371/journal.pone.0228463)

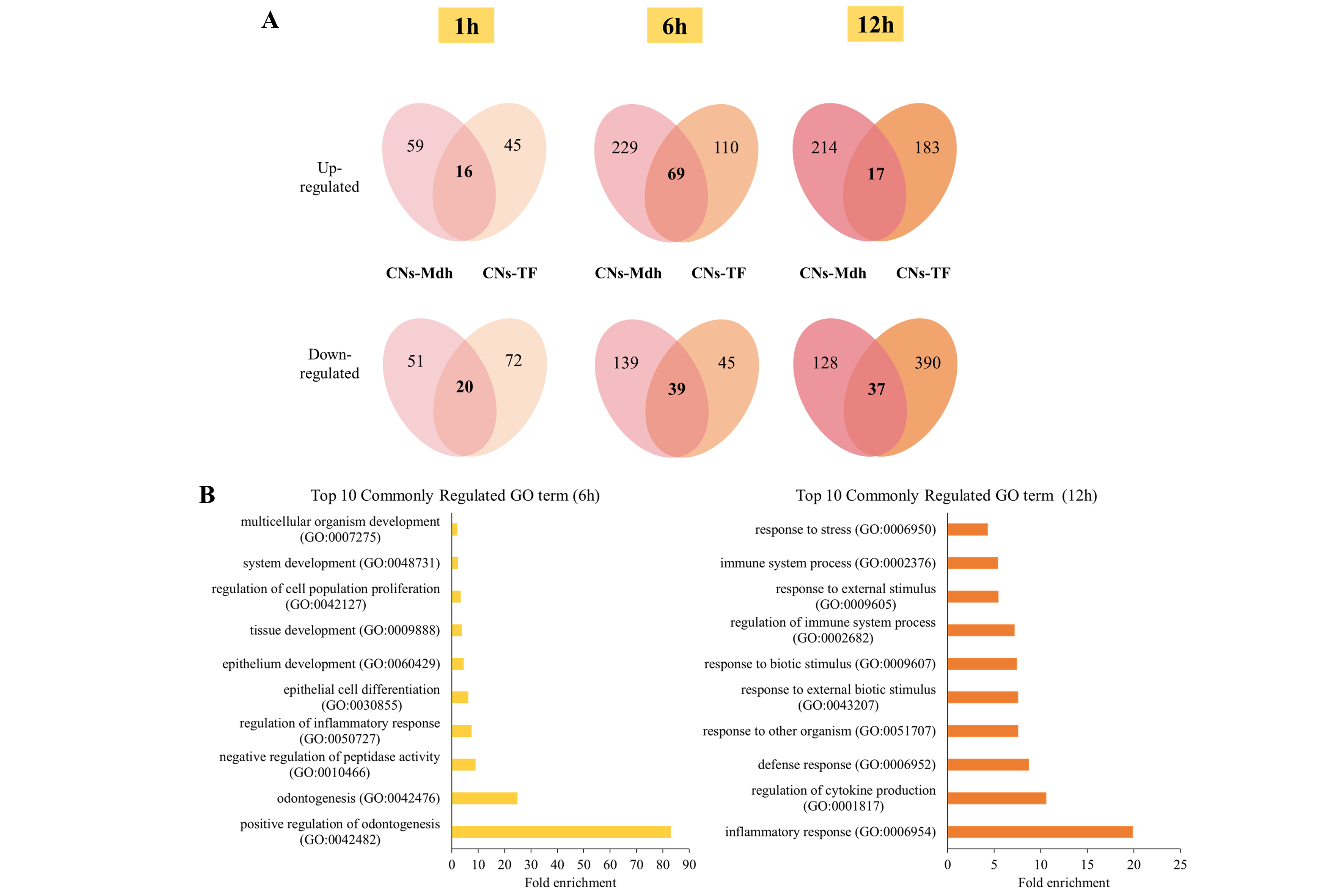

Supplement: S1 Fig — (A) Venn diagram of DEGs from only the CN-Mdh- and CN-TF-immunized groups compared to the CNs-immunized group. (B) Gene Ontology enrichment analysis using commonly regulated genes from only the CN-Mdh and CN-TF-immunized groups. (TIF) [file pone.0228463.s002.tif]

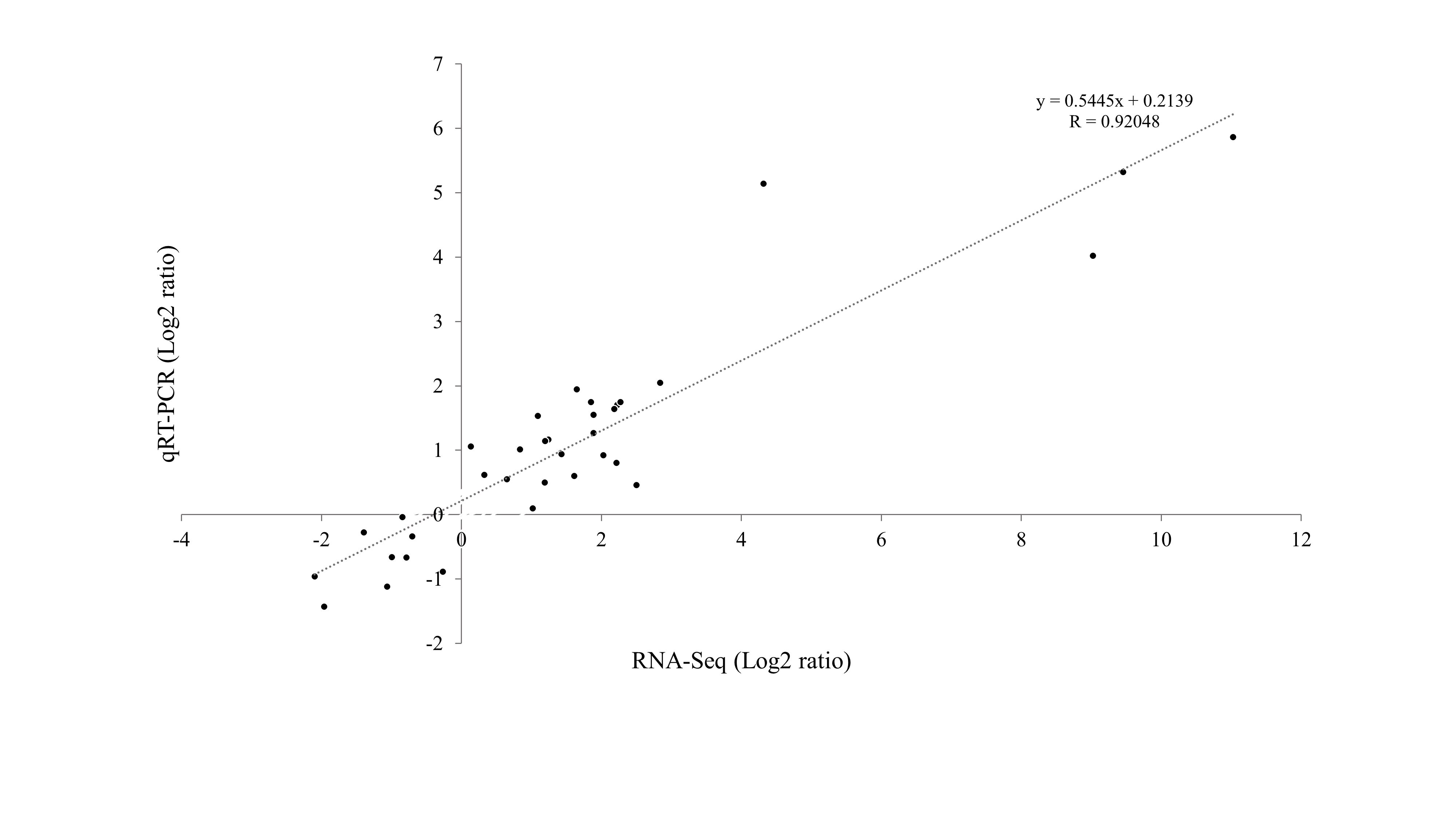

Supplement: S2 Fig — The six validated genes (Il1rn, Batf, Tlr4, Il6, Il23a and Tnf) were important for the inflammatory response and differentiation of Th17 cells. (TIF) [file pone.0228463.s003.tif]
