# Supplementary material for: Induction of systemic immunity through nasal-associated lymphoid tissue (NALT) of mice intranasally immunized with Brucella abortus malate dehydrogenase-loaded chitosan nanoparticles
Source: PLoS One. 2020 Feb 6;15(2):e0228463. doi: 10.1371/journal.pone.0228463 (PMC7004331; doi:10.1371/journal.pone.0228463)
Supplement: S1 Raw image — (PDF) [file pone.0228463.s004.pdf]

Uncropped and unadjusted images for SDS-PAGE and Western blot of Fig 1

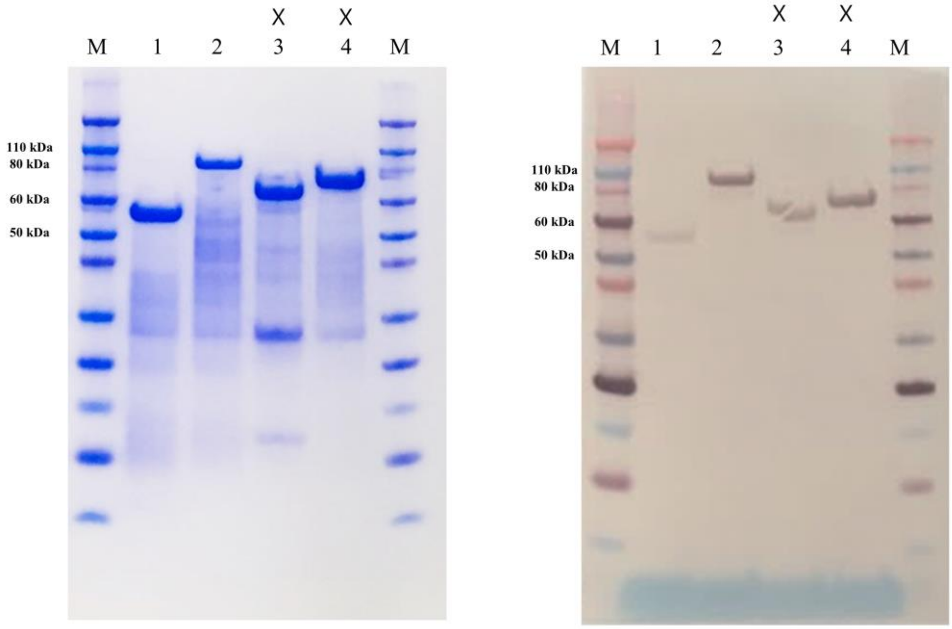

Corresponds to Fig 1. SDS-PAGE and Western blotting. Lane M: Molecular size standard, Lane 1: Control, TF (53 kDa), Lane 2: Mdh (92.71 kDa), Lanes 3-4: Other proteins not included in the final figure.
