# Supplementary material for: Induction of systemic immunity through nasal-associated lymphoid tissue (NALT) of mice intranasally immunized with Brucella abortus malate dehydrogenase-loaded chitosan nanoparticles
Source: PLoS One. 2020 Feb 6;15(2):e0228463. doi: 10.1371/journal.pone.0228463 (PMC7004331; doi:10.1371/journal.pone.0228463)
Supplement: S1 Table — (DOCX) [file pone.0228463.s005.docx]

**S1 Table. Primers used for qRT-PCR**

| Genes | F | R | Reference |
| --- | --- | --- | --- |
| *Batf* | CCAGAAGAGCCGACAGAGAC | GAGCTGCGTTCTGTTTCTCC | [45] |
| *Il1rn* | ACCTTCCCACAGCGGCTCCACATT | TTGTCAAGAAGCAGAGGTTTACAG | [45] |
| *Il23a* | CTGCTTGCAAAGGATCCACC | TTGAAGCGGAGAAGGAGACG | [46] |
| *Il6* | CTCTGCAAGAGACTTCCA | AGTCTCCTCTCCGGACTT | Custom |
| *Tlr4* | GTGCCAATTTCATGGGTCT | CATCGAAGTCAATTTTGGTGTT | Custom |
| *Tnf* | CAACGCCCTCCTGGCCAACG | TCGGGGCAGCCTTGTCCCTT | [47] |
| *Gapdh* | CCCCAGCAAGGACACTGAGCAAG | TGGGGGTCTGGGATGGAAATTGTG | [48] |

45. Akitsu A, Ishigame H, Kakuta S, Chung SH, Ikeda S, Shimizu K, et al. IL-1 receptor antagonist-deficient mice develop autoimmune arthritis due to intrinsic activation of IL-17-producing CCR2(+)Vgamma6(+)gammadelta T cells. Nature communications. 2015;6:7464. Epub 2015/06/26. doi: 10.1038/ncomms8464. PubMed PMID: 26108163; PubMed Central PMCID: PMCPMC4521288.

46. Sato K, Suematsu A, Okamoto K, Yamaguchi A, Morishita Y, Kadono Y, et al. Th17 functions as an osteoclastogenic helper T cell subset that links T cell activation and bone destruction. The Journal of experimental medicine. 2006;203(12):2673-82. Epub 2006/11/08. doi: 10.1084/jem.20061775. PubMed PMID: 17088434; PubMed Central PMCID: PMCPMC2118166.

47. Das S, Seth RK, Kumar A, Kadiiska MB, Michelotti G, Diehl AM, et al. Purinergic receptor X7 is a key modulator of metabolic oxidative stress-mediated autophagy and inflammation in experimental nonalcoholic steatohepatitis. American journal of physiology Gastrointestinal and liver physiology. 2013;305(12):G950-63. Epub 2013/10/26. doi: 10.1152/ajpgi.00235.2013. PubMed PMID: 24157968; PubMed Central PMCID: PMCPMC3882442.

48. Cha SB, Lee WJ, Shin MK, Jung MH, Shin SW, Yoo AN, et al. Early transcriptional responses of internalization defective Brucella abortus mutants in professional phagocytes, RAW 264.7. BMC genomics. 2013;14:426. Epub 2013/06/28. doi: 10.1186/1471-2164-14-426. PubMed PMID: 23802650; PubMed Central PMCID: PMCPMC3716731.
