# Supplementary material for: Induction of systemic immunity through nasal-associated lymphoid tissue (NALT) of mice intranasally immunized with Brucella abortus malate dehydrogenase-loaded chitosan nanoparticles
Source: PLoS One. 2020 Feb 6;15(2):e0228463. doi: 10.1371/journal.pone.0228463 (PMC7004331; doi:10.1371/journal.pone.0228463)
Supplement: Attachment — Submitted filename: Response to Reviewers.docx [file pone.0228463.s006.docx]

**Comments from the editor and reviewers:**

I would like to thank for the comments from reviewer. Comments from reviewer make improve our manuscript. We revised our manuscript based the comments and tried our best to revise.

**- Reviewer's comments:**

**Reviewer #1**

1. Overall, the manuscript is well written and the experimental design is straightforward and easy to understand. However, the authors are over-reaching in their conclusions based on the available data and there are some immunological concepts that need to be revised throughout the manuscript. The authors should consider two key points: 1) Chitosan has natural adjuvant activity in that it induces dendritic cell activation to promote TH1 responses, mediated through STING, which doesn’t quite fit with the authors conclusion of a TH2-type of response, nor does the data really support the conclusion that a TH2 response has been induced; and 2) For *Brucella* clearance, induction of a TH1 response is necessary, so induction of a TH2 response in not a desirable outcome from a vaccine against brucellosis. The authors should consider this and address it in their discussion.

**Answer.**

We appreciate your valuable comment. As your opinion, we might have jumped to the conclusion. In our previous study, intranasal immunization of CNs-Mdh induced systemic mucosal immunity with Th2-related response at 6 wpi [1]. In this study, we aimed to observe whether our antigens properly activate the NALT, the target site of the immunization, and the antibody is produced after intranasal immunization of CNs-Mdh. The pathway analysis showed that the DEGs were significantly enriched in multiple inflammatory pathways including “IL-6 Signaling”, "Dendritic cells maturation" and “HMGB1 Signaling” in CNs-Mdh-immunized group. At the 2 wpi, the production of specific IgG1 and IgG2a in sera indicates that a mixed Th1-Th2 response were elicited by CNs-Mdh. Taken together, we concluded that immunocompetent cells in NALT activated IL-6 signaling pathway and DCs maturation in early time point and elicited a mixed Th1-Th2 response in early stages, then finally differentiated into Th2 by CNs-Mdh. Therefore, we revised the concept of the manuscript (Lines 451-459).

Until now, the vaccines inducing cellular immunity have been known to be effective for intracellular bacteria. However, the subunit vaccines which induce not only humoral immunity but also complex immune response are being suggested as a replacement to the existing vaccines recently [2]. In addition, protective immunity of host against *Brucella abortus* is mediated by both cellular and humoral immune responses. A recent study showed that IL-6 promotes *Brucella abortus* clearance in macrophages and CD8+ T cell differentiation, priming the Th1 response during *Brucella* infection [3]. The live attenuated vaccine strains for brucellosis, S19 and RB51 are known to induce strong cellular immunity, but the antibody profile observed in both regimens were reported as predominantly IgG1 [2]. This discrepancy suggests that the course of infection and immune protection against *Brucella* involves complicated balance between Th1 and Th2 related immune response.

We tried to suggest not only the new antigen delivery system, but also a convenient method to induce systematic IgG and IgA immune responses with relatively small amount of *Brucella* antigen. In addition, a functional assessment of this antibody is planned for further study by modulating infection. We believe that this study could contribute to the development of subunit vaccine. Finally, the sentences about protective immunity against *Brucella* were added in the discussion (Lines 443-449). Thank you.

2. Line 306-307: “….active movement of leukocytes, phagocytes, granulocytes….indicating that the antigen recognition was activated.” Migration of these cells is not indicative of antigen recognition, but rather inflammation. Sentence should be revised.

**Answer.**

The sentence has been amended as recommended (Lines 309-310).

3. Line 329-330. Referring to IL-1beta, IL-6, NF-kB, etc. as inducers of Th2 responses needs revision. The mentioned molecules are indeed involved in the proinflammatory response, but not necessarily specific drivers of Th2 differentiation.

**Answer.**

The sentence has been amended as recommended (Lines 330-332).

4. Line 439-440: “IL-6 has been indicated as a key cytokine for the differentiation of Th2 cells in both mice and humans.” IL-6 is involved in both TH1 and TH2 responses, and at the early stages that these samples are being analyzed, the skewing of adaptive immune responses cannot be determined. Sentence should be revised.

**Answer.**

The sentence has been amended as recommended (Line 440).

5. Line 480-482: This is overinterpretation of the data. The work simply shows that upregulation of HMGB1, IL-6, and DC maturation at the early time points after immunization may be correlated to increased IgA production at 2 wpi. These experiment were not designed to show causation.

**Answer.**

We appreciate your valuable comment. As the relationship between innate immune pathway and production of IgA is still not well defined, we found other studies about HMGB1 signaling, IL-6 signaling, DCs maturation and genes related to production of IgA as we described in the manuscript (Lines 465-487). Since the secretory IgA-mediated mucosal immunity is not always induced through mucosal immunization at local site, we thought that activated pathways and gene network induced by CNs-Mdh were related to production of IgA. However, as your opinion, we have not examined causative relationship and we revised the sentence (Lines 489-490). Thank you.

6. The IgG and IgA data is not very impressive. While there is statistical significance, the levels of total IgG, IgG1 and IgG2a are not very high when compared to the rest of the experimental groups. Perhaps looking at later time points would have helped enhance that response. Additionally, the antibody data does not support the induction of a TH2 response, rather, it appears mixed. Other parameters such as cytokine production from recall responses would have been a better approach to demonstrate the type of immune response that was induced by this type of immunization.

**Answer.**

We appreciate for your valuable comment. As we described above, we revised the concept of the manuscript. In addition, here is the reason we looked the early time point; since we observed humoral responses at 4wpi and 6 wpi in our previous study [1], we aimed to observe whether our antigens properly activate the NALT and the antibody is produced after intranasal immunization of CNs-Mdh in this study. We regarded 2 weeks long enough, considering the production of IgG and IgA after antigen recognition in NALT and inducing systemic response. It would be more helpful to observe enough cytokines produced by recall responses, but considering high prices of RNA-seq and the number of samples, we had to selectively observe the transcriptomic data and antibody. I hope your understanding on our current limitation on resources.

7. It should be noted that there is no mention of antibody data in the introduction of the manuscript, yet the authors utilize the antibody data to tie in their transcriptomic data to an immunological phenotype. It is in the trying connect these two pieces of data (i.e. gene expression and humoral responses) that the manuscript loses its strength, as these data are not congruent with one another.

**Answer.**

We appreciate for your valuable comment. We added sentence explaining the reason we have investigated the titer of antibody in introduction (lines 68-72; 80-81).

[References]

1. Soh SH, Shim S, Im YB, Park HT, Cho CS, Yoo HS. Induction of Th2-related immune responses and production of systemic IgA in mice intranasally immunized with Brucella abortus malate dehydrogenase loaded chitosan nanoparticles. Vaccine. 2019;37(12):1554-64.

2. Dorneles EM, Lima GK, Teixeira-Carvalho A, Araujo MS, Martins-Filho OA, Sriranganathan N, Qublan H, Heinemann MB, Lage AP, 2015. Immune Response of Calves Vaccinated with *Brucella abortus* S19 or RB51 and Revaccinated with RB51. PLOS one 10, e0136696.

3. Hop HT, Huy TXN, Reyes AWB, Arayan LT, Vu SH, Min W, Lee HJ, Kang CK, Kim DH, Tark DS, Kim S, 2019. Interleukin 6 (IL-6) promotes *Brucella abortus* clearance by controlling bactericidal activity of macrophages and CD8(+) T cell differentiation. Infect Immun. 18;87(11).
